# Overcoming the Limitations of Fragment Merging: Rescuing a Strained Merged Fragment Series Targeting *Mycobacterium tuberculosis* CYP121

**DOI:** 10.1002/cmdc.201300219

**Published:** 2013-06-20

**Authors:** Sean A. Hudson, Sachin Surade, Anthony G. Coyne, Kirsty J. McLean, David Leys, Andrew W. Munro, Chris Abell

**Affiliations:** ^1^ Department of Chemistry, University of Cambridge, Lensfield Road, Cambridge, CB2 1EW (UK) http://www‐abell.ch.cam.ac.uk/; ^2^ Department of Biochemistry, University of Cambridge, 80 Tennis Court Road, Cambridge, CB2 1GA (UK); ^3^ Manchester Institute of Biotechnology, University of Manchester, 131 Princess Street, Manchester, M1 7DN (UK); ^4^ Present Address: Department of Pharmaceutical Chemistry and Cellular & Molecular Pharmacology, University of California San Francisco (UCSF), San Francisco, CA 94158 (USA)

**Keywords:** conformation analysis, cytochromes, drug discovery, fragment‐based, tuberculosis

## Abstract

**Freedom to merge:** A combination of crystal structure examination and in silico predictions made it possible to overcome the conformational limitations of fragment merging and escape the internal strain in a series of weakly binding merged fragments that target *M. tuberculosis* CYP121. The insights attained provide a new perspective and guide for prioritizing synthetic efforts toward fragment merging in future and ongoing fragment‐based ligand discovery campaigns.

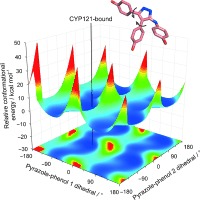

The fragment‐based approach is a firmly established paradigm for developing small‐molecule ligands as chemical tools and leads for drug development.^1^ At its heart, this powerful methodology involves the structure‐guided design and synthesis of potent ligands from weak binding low‐molecular‐weight fragment molecules (typically <250 Da).^1^ There are three main strategies for the elaboration of initial fragment hits:1a 1) fragment growing, in which fragments are grown structurally into new unexplored regions within/around their binding site; 2) fragment linking, where two or more fragments that bind in close proximity within the binding pocket are covalently linked; and 3) fragment merging, in which the substructural components of fragments that overlap in the binding cavity are fused together. Although fragment merging represents a particularly elegant solution, the strategy remains relatively unexplored in fragment‐based ligand discovery (FBLD) campaigns.^1^ Rather, hybrid merged molecules are far more common, formed by combining fragment hits with elements from a known larger substrate, cofactor, or inhibitor.1a, 2

We recently presented the first successful fragment‐based approach to targeting cytochrome P450 enzymes (CYPs), which highlighted significant limitations with fragment merging in regards to maintaining conformational freedom in the elaborated molecules.^3^ In this program against the *Mycobacterium tuberculosis* (*Mtb*) cytochrome P450 CYP121 (the gene for which is essential for *Mtb* viability), overlapping fragments were found in both heme‐binding and non‐heme‐coordinating active‐site positions. Their elaboration via a fragment merging strategy proved highly successful for the heme‐binding fragments, but this was not recapitulated by compounds based on the non‐heme‐coordinating triazolylphenol fragment **1** (Figure 1, fragment **1**­ *K*_D_=∼1.7 mm, merged fragment series **2**–**4**­ *K*_D_=∼1–3 mm). Using the representative weak merged fragment **4**, it was shown by crystallographic and quantum mechanical (QM) computational analysis that there is a large conformational energy barrier necessary for the merged compounds to bind CYP121, due to steric clash of their phenol *ortho*‐hydrogen atoms (see Figure 1 highlighted red). The primary CYP121 interactions that were identified to sandwich the phenol pair of **4** in its conformationally strained state were a combination of: 1) a hydrogen bond between Asn85 and the hydroxy group of the phenol pointing toward the heme; 2) stacking of the second phenol between Phe168 and Trp182 (∼4 Å edge‐to‐face), with its hydroxy group protruding into the water network of the putative substrate entry channel; and 3) an offset π‐stack between the triazole ring and Phe168 (∼3.7 Å between the planes). The triazole nitrogen atoms could hydrogen bond to either Thr77 or Gln385, but there was no clear preference, and both interactions were modeled for the parent fragments.

**Figure 1:**
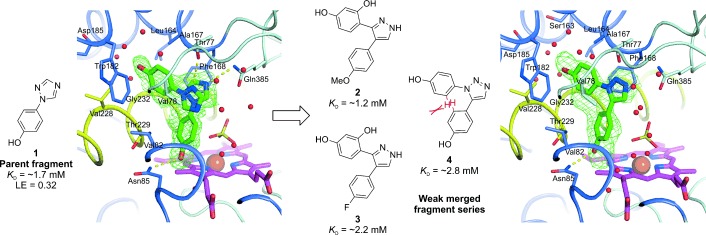
Previous fragment‐merging strategy for the non‐heme‐coordinating triazolylphenol fragment **1** binding *Mtb* CYP121.^3^ The CYP121 active‐site complex with **1** and **4** is shown (PDB IDs: 4G47 and 4G2G, respectively). QM‐based conformational energetic calculations revealed that significant internal strain is introduced in the representative weak merged fragment **4** on binding CYP121 due to its *ortho*‐hydrogen atoms on opposite phenols clashing with each other to one side of the triazole ring (illustrated red).

Herein we report how a combination of crystal structure examination and in silico predictions made it possible to overcome the conformational limitations of fragment merging and escape the internal strain in the weakly binding merged fragment series **2**–**4**. Thorough investigation of the crystallographic active‐site complex, coupled with virtual screening, led to new structural scaffolds that were anticipated to alleviate the internal strain by subtly altering the ligands’ binding pose using a double hydrogen bond with a nearby Gln385 residue. Synthesis and rational selection from a high‐throughput screening library led to the discovery of a novel non‐heme‐coordinating aminopyrazole ligand with over 185‐fold greater affinity (*K*_D_=15 μm) than the closest original conformationally strained merged fragment. The lead is the first high‐affinity ligand for the essential *Mtb* CYP121 enzyme that does not feature a generic CYP heme‐coordinating interaction. It is also the highest‐affinity ligand developed using fragment‐based approaches against any CYP. Based on the findings, we derive conclusions about the overall limitations and requirements for fragment merging strategies in FBLD. The insights attained provide a new perspective for prioritizing synthetic efforts toward fragment merging in future and ongoing FBLD campaigns.

First, to identify a solution to removing internal strain in the representative merged fragment **4**, its crystallographic binding pose was examined along with the surrounding region of the CYP121 active site (see Figure 1). A significant contributing factor to its *ortho*‐hydrogen clash was the rigid constraint of the phenol hydrogen bonding to Asn85, which lies tightly packed against the Val82 and Thr229 residues. Previous conformational energy calculations suggested that a free rotation of this phenol by as little as 20° could halve the internal conformational strain in the CYP121‐bound pose.^3^ A clear possibility to enable such rotation was presented by the neighboring Gln385 residue on the opposite side of the narrow active‐site channel (see Figure 1). We hypothesized that if an interaction with Gln385 could be strengthened (e.g., by using a double hydrogen bond)^4^ via subtle structural modification of **4**, it could shift the restricted phenol sufficiently away from Val82 and Thr229 to return its rotational freedom, while still enabling **4** to maintain the other important interactions discussed above.

To find new structural scaffolds that could promote a dual hydrogen bond with the Gln385 side chain, a focused virtual screen was performed using GOLD docking for 31 biphenol analogues of **4** with various five‐membered aromatic heterocycle motifs (structures in Supporting Information figure S1). The heterocycles had an additional hydrogen bond donor (or *protonated amine*) positioned for potential interaction with the carbonyl of the Gln385 amide. Twenty‐eight of the 31 analogues docked in the same general pose as **4** (overlaid docking poses in Supporting Information figure S2 a), and the new heterocycles found active‐site positions somewhere between the original site occupied by **4** and immediately adjacent to Gln385. Those scaffolds that showed the strongest interaction with Gln385 (i.e., nearest to the optimum hydrogen bond distance and directionality as defined by the GoldScore function) were those featuring an arylamine substituent, 3‐aminopyrazole (Figure 2 a, compound **5**) and amino‐1,2,3‐triazoles (structures boxed in Supporting Information figure S1), in which the ring nitrogen atom and arylamine formed the intended double hydrogen bond with the Gln385 amide. This dual donor/acceptor hydrogen bonding ability between 3‐aminopyrazoles and amides has been used previously to stabilize the β‐strand conformation of small peptides.^5^ It is also worth noting that a simpler pyrazole motif (no amine substituent) had been tested experimentally before as part of the weak merged fragments **2** and **3**, as well as a phenoxypyrazole ligand **s1** (*K*_D_=500±200 μm),^3^ where this analogue interacted with Thr77 rather than Gln385 (see Supporting Information figure S4). The phenols of the docked analogues were found in a range of rotational conformations (see Supporting Information figure S2 a), but none showed significant *ortho*‐hydrogen clash. However, when **4** was docked it also did so in a lower‐energy conformation and closer to Gln385 than the experimentally observed position (see Supporting Information figure S2 b).

**Figure 2:**
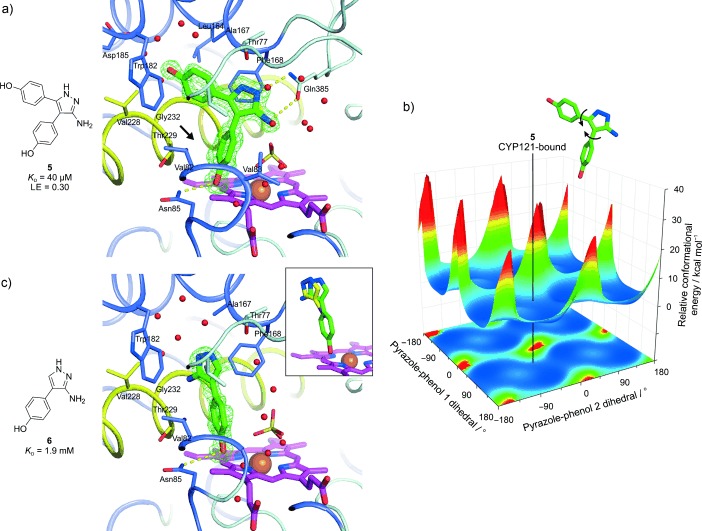
a) X‐ray crystal structure at 1.35 Å resolution of **5** in complex with CYP121, with its associated binding affinity determined by ITC and calculated LE (kcal mol^−1^ NHA^−1^). The phenol (rotated relative to the corresponding group in **4**; see Figure 1) responsible for mitigating steric hindrance is clearly indicated (arrow). Side chains and water/sulfate molecules within 5 Å of the bound ligand are shown as thin sticks and red spheres, respectively. Direct ligand–CYP121 hydrogen bonds are represented as dashed yellow lines. The *F*_o_−*F*_c_ omit electron density map associated with the ligand is shown as a green mesh contoured at 3 *σ*. b) Computed conformational energy landscape of **5** for all rotations of its aminopyrazole–phenol dihedral angles (force field OPLS‐2005 based methods,^9^ solvent: water). High‐energy peaks (red) correspond to full steric clash between the phenol *ortho*‐hydrogen atoms. The low‐energy dihedral positions for CYP121‐bound **5** are indicated (no *ortho*‐hydrogen steric clash). QM calculations (QM method: DFT(b3lyp), QM basis: 6‐31g**, gas phase) indicate no significant internal strain for the protein‐bound pose relative to the predicted ground‐state conformation in the gas phase (0.4 kcal mol^−1^). This calculated energy difference is 21‐fold lower than for **4**.^3^ c) CYP121 active‐site complex with **6** from a 1.35 Å crystal structure, with its associated *K*_D_ value determined by ITC. The view and colors are the same as those described for panel a). The inset in panel c) shows an overlay of the comparative CYP121 binding positions of **6** and iodopyrazole from the 1.8 Å co‐crystal structure by Leys et al.^6^ (PDB ID: 1N4G).

The virtual screening results suggested that the 3‐aminopyrazole **5** would make the closest interaction with Gln385. Consequently, synthetic routes were derived to it (reaction scheme in Supporting Information figure S3 a), as well as its single‐phenol analogues **6** and **7** (Table 1 and Supporting Information figure S3 b,c).

**Table 1:** The publisher did not receive permission from the copyright owner to include this object in this version of this product. Please refer either to the publisher's own online version of this product or the printed product where one exists.

The CYP121 binding affinities (*K*_D_ values) of the aminopyrazoles **5**–**7** were determined by isothermal titration calorimetry (ITC) using direct titrations of ligand into CYP121, and their ligand efficiencies (LEs) were calculated from the *K*_D_ values (Figure 2 and Table 1). Remarkably, **5** had a *K*_D_ of 40 μm, 70‐fold greater than the original strained merged fragment **4**, and a correspondingly high LE of 0.30 kcal mol^−1^ NHA^−1^ (NHA=non‐hydrogen atom). Analogues **6** and **7**, on the other hand, did not bind with notably higher affinity than the analogous triazolylphenol fragment **1** (*K*_D_: 1.9, 1.3, and 1.7 mm for **6**, **7**, and **1**, respectively).

CYP121 crystals were individually soaked with aminopyrazole **5** and its analogues, leading to the successful determination of structures of **5** and **6** in complex with CYP121, both at 1.35 Å resolution (Figure 2 a,c). As predicted, compound **5** successfully positions to make a double hydrogen bond with the Gln385 side chain through its aminopyrazole. It also maintains all prominent interactions with the protein identified by **4**, including one phenol hydrogen bonding to Asn85 and the other aromatic stacking between Phe168 and Trp182. The Gln385‐promoted interaction moves **5** sufficiently far from lying flat against Val82 and Thr229, such that there is a notable rotation in the originally constrained/restricted phenol adjacent to these residues (corresponding azole–phenol dihedral angles of 30° vs. 65° for **4** and **5**, respectively; see Figures 1 and 2 a). In silico QM calculations were performed for the conformational energetics of **5**, which show that this rotation clearly alleviates the steric clash of the phenol *ortho*‐hydrogen atoms observed for **4** (Figure 2 b).

Analogue **6** adopts an unexpectedly different binding mode from that of **5**, with its aminopyrazole instead in the small substrate entry channel region stacked between Phe168 and Trp182, as for one phenol of **5** (Figure 2 c). Its aminopyrazole makes no clear interactions with the protein and points into the exterior water network of the substrate entry cavity. This is the same pyrazole binding mode observed previously for iodopyrazole binding to CYP121 (Figure 2 c, inset).^6^ The iodine atom binds in the same orientation as the phenol ring of **6**, buried between the hydrophobic side chains of Val78, Phe168, Thr229, and Ala233. The phenol maintains the same hydrogen bond with Asn85 as **5**, despite the change in trajectory. Comparison of the overall binding poses of **5** and **6**, and also taking into account the weak affinity of both analogues **6** and **7**, suggests that two phenols are the minimal requirement to maintain the potent aminopyrazole interaction/binding pose with Gln385.

In an attempt to further improve the potency of **5** and explore new chemical space around the lead, we searched for compounds with: 1) a similar core scaffold to that of the lead and 2) a diverse selection of attached substructures that could probe and be accommodated within the large CYP121 water‐filled active‐site to enhance binding. New moieties were selected based on visual inspection of the **5**–CYP121 crystal structure (see Figure 2 a), to the extent of matching polar/nonpolar groups to similar active‐site regions/pockets. Although interfacial waters are known to form energetically stable hydrogen bonding networks that are sometimes more favorable than direct ligand–protein contacts,^7^ the CYP121 interstitial waters were not explicitly considered in the selection process simply due to the overall magnitude of the water network (∼1350 Å^3^). In total, six analogues were acquired (Table 1 compounds **9**–**14**). The analogues **9**–**14** all maintained a similar 6‐5‐6 ring system to **5**, and comprised new vectors of chemical growth extending primarily from the five‐membered heterocycle or phenol that points toward the heme. In addition, a simple direct analogue of **5** was synthesized with an *ortho*‐hydroxy group installed on one phenol (Table 1 compound **8**), in an attempt to capture the possible extra hydrogen bond with Thr77 identified by the aforementioned phenoxypyrazole ligand **s1** (see Supporting Information figure S4).

Analogues **8**–**14** were screened against CYP121 for binding, and their affinity determined in individual ITC experiments injecting ligand into 50 or 100 μm CYP121 (Table 1). While five out of seven showed no binding (heat of ligand dilution only), two prominent hits were identified—**8** (*K*_D_=180 μm) and **9** (*K*_D_=15 μm)—the latter representing over 185‐fold higher potency than the original strained merged fragment **4**.

X‐ray crystal structures at 1.40 and 1.95 Å resolution of **8** and **9** in complex with CYP121 were successfully determined through crystal soaking (Figure 3). Both ligands recapitulated the general binding pose of **5**, with their aminopyrazoles forming the clear double hydrogen bond with Gln385. Compound **8** closely mirrors the binding pose of **5**, and its additional *ortho*‐hydroxy group makes the intended hydrogen bond with Thr77 despite its weakened affinity (see above). The surprising loss of affinity associated with the *ortho*‐hydroxy group in **8** could be the result of a combination of an alteration in the water network around the substituted phenol (Figure 3 a, c.f. Figure 2 a), and loss of an internal hydrogen bond between the pyrazole N/NH and the phenol *ortho*‐hydroxy group of **8** on binding CYP121 (the corresponding pyrazole/phenol in protein‐bound **8** are out‐of‐plane with a dihedral angle of 35°). Tightly angled intramolecular hydrogen bonds involving five‐membered rings and their substituents have been detailed previously,^8^ and the CYP121‐bound pose of the original phenoxypyrazole ligand **s1** from which **8** was designed (no Gln385 interaction) also has in‐plane pyrazole/phenol rings (see Supporting Information figure S4).

**Figure 3:**
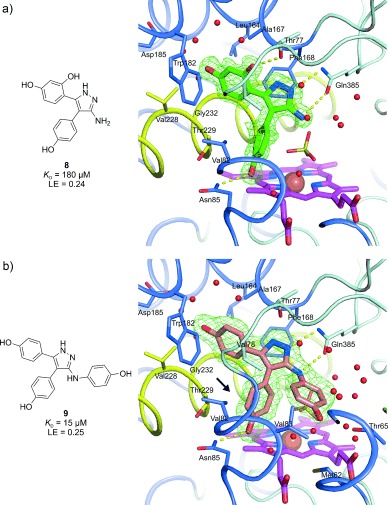
The CYP121 active‐site complex with a) **8** and b) **9** from 1.40 and 1.95 Å X‐ray crystal structures, respectively, and with their associated *K*_D_ values determined by ITC and calculated LE. The view and colors are the same as those described in Figure 2. The rotated phenol group of **9** slotted between Val82 and Thr229 is highlighted (arrow).

In the lead compound **9**, the third —NHR phenol protrudes into the large CYP121 water‐filled active‐site cavity, but does not appear to make significant interactions directly with CYP121, and instead only forms hydrogen bonds to the interstitial water network. This may explain its decrease in LE (0.30 vs. 0.25 kcal mol^−1^ NHA^−1^). There is also a notably different rotation for the phenol closest to the heme between **9** and **5** (Figure 3 b, c.f. Figure 2 a). The central phenol in **9** lies slotted between the Val82 and Thr229 residues (corresponding pyrazole–phenol dihedral angles of 65° vs. −45° for **5** and **9**, respectively). This could be a consequence of steric hindrance from the extra —NHR phenol on binding, or due to the increased hydrogen bonding proximity of the entire structure to Gln385 (the hydrogen bonding distance to the Gln385 carbonyl by **9** is 3.1 Å compared with 3.4 Å for **5**), moving it even further away from Val82 and Thr229. Energetic calculations were performed for **9** as per **5** (see Supporting Information figure S5), and as expected, the phenols directly attached to the pyrazole remain sufficiently out of plane with each other to escape any phenol *ortho*‐hydrogen clash.

In this study an illustrative stepwise approach was used to assess and overcome the conformational limitations of fragment merging and rescue the series of low‐affinity merged fragments **2**–**4** hindered by conformational strain on binding the *Mtb* CYP121 active‐site. Based on our experiences using FBLD against CYP121 (summarized in Table 2), we can draw some conclusions about the overall limitations and requirements for fragment merging strategies. The observed success or failure of fragment merging appears generally ordered with the average distance between the common atoms shared by the two parent fragments. It is logical that the greater the atomic overlap of the initial fragment hits, the more likely the merged fragments will be to recapitulate their parent fragments binding pose and interactions. The analysis provides an estimate that the maximum average distance between shared atoms required for successful fragment merging may be between 0.4 and 1.0 Å. However, this situation also becomes increasingly complicated in comparing the failed fragment set 3 (Table 2). While set 3 had greater atomic overlap than set 2, it is the only one in which an entirely different substructural moiety was present between the fragments in the same binding loci (i.e., phenol vs. carboxylate) and one had to be sacrificed, along with its binding interactions, to build the merged fragments, which ultimately failed. In addition to the closeness of the common atoms/moieties, the experiences with fragment set 2 clearly indicate that considerable importance should be given to estimating the magnitude of any internal strain that would be imposed on elaborated molecules, in order to maintain the binding pose of their parents. Our methodology presented herein for calculating strain (see Supporting Information, experimental methods) is easily applicable to any ligand–target system. These findings provide a guide for the assessment and prioritization of synthetic investment toward merging molecules in FBLD campaigns.

**Table 2:** The publisher did not receive permission from the copyright owner to include this object in this version of this product. Please refer either to the publisher's own online version of this product or the printed product where one exists.

## Supporting Information

All biological, chemical, and computational experimental methods are given in the Supporting Information.

## Supplementary Material

As a service to our authors and readers, this journal provides supporting information supplied by the authors. Such materials are peer reviewed and may be re‐organized for online delivery, but are not copy‐edited or typeset. Technical support issues arising from supporting information (other than missing files) should be addressed to the authors.

miscellaneous_information
