# Supplementary material for: Overcoming the Limitations of Fragment Merging: Rescuing a Strained Merged Fragment Series Targeting Mycobacterium tuberculosis CYP121
Source: ChemMedChem. 2013 Jun 20;8(9):1451–6. doi: 10.1002/cmdc.201300219 (PMC4281926; doi:10.1002/cmdc.201300219)
Supplement: Supplementary file 1 — miscellaneous_information [file cmdc-8-1451-s1.pdf]

CHEMMEDCHEM
Chemistry & Drug Discovery

Supporting Information

© Copyright Wiley-VCH Verlag GmbH & Co. KGaA, 69451 Weinheim, 2013

Overcoming the Limitations of Fragment Merging: Rescuing a Strained Merged Fragment Series Targeting *Mycobacterium tuberculosis* CYP121

Sean A. Hudson,[a, d] Sachin Surade,[b] Anthony G. Coyne,[a] Kirsty J. McLean,[c] David Leys,[c] Andrew W. Munro,[c] and Chris Abell*[a]

cmdc_201300219_sm_miscellaneous_information.pdf

Supporting Information
Table of Contents

Supplementary Figures and Tables **2**

Figure S1. 2

Figure S2. 3

Figure S3. 3

Figure S4. 4

Figure S5. 4

Table S1. 5

Experimental Methods **6**

Materials 6

Virtual screening 6

Expression and purification of *Mtb* CYP121 7

Isothermal titration calorimetry (ITC) 7

X-ray crystallography 7

In silico conformational energetic calculations 7

Organic synthesis 8

References **13**

Supplementary Figures and Tables

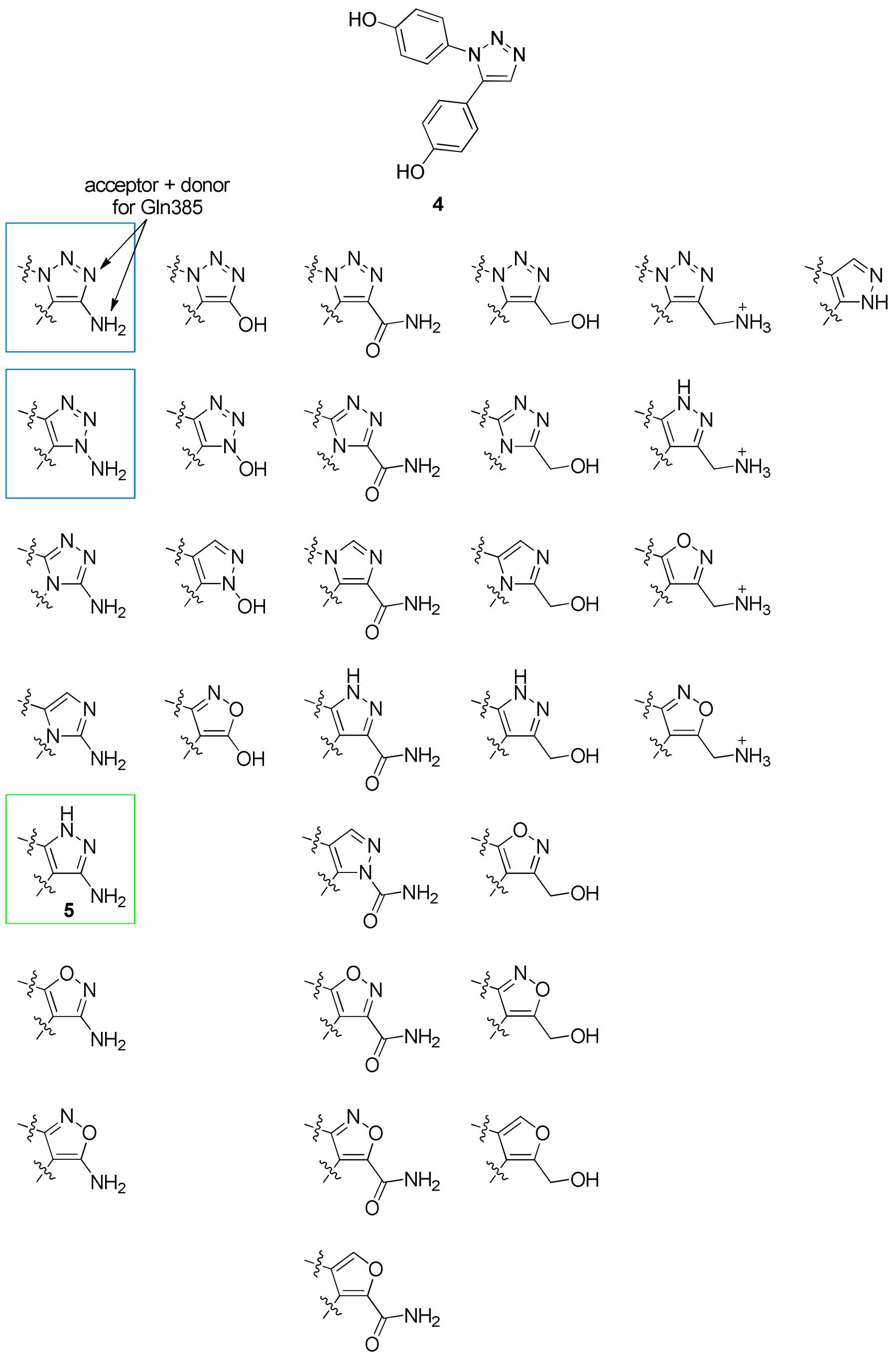

Figure S1. The thirty-one biphenol analogs of **4** (varying heterocycle motifs) rationally selected for virtual screening to promote an interaction with the CYP121 Gln385 side chain. All the structures had been previously reported featuring biaryl substituents in the SciFinder chemical databank (https://scifinder.cas.org/). They are drawn in their predominant protonation state at pH 5-6 (CYP121 crystallization pH). The top three scaffolds predicted to make the closest proximity interaction with Gln385 (double H-bond with the amide side chain) were those featuring an arylamine substituent, the 3-aminopyrazole (green box, compound **5**) and amino-1,2,3-triazoles (blue box).

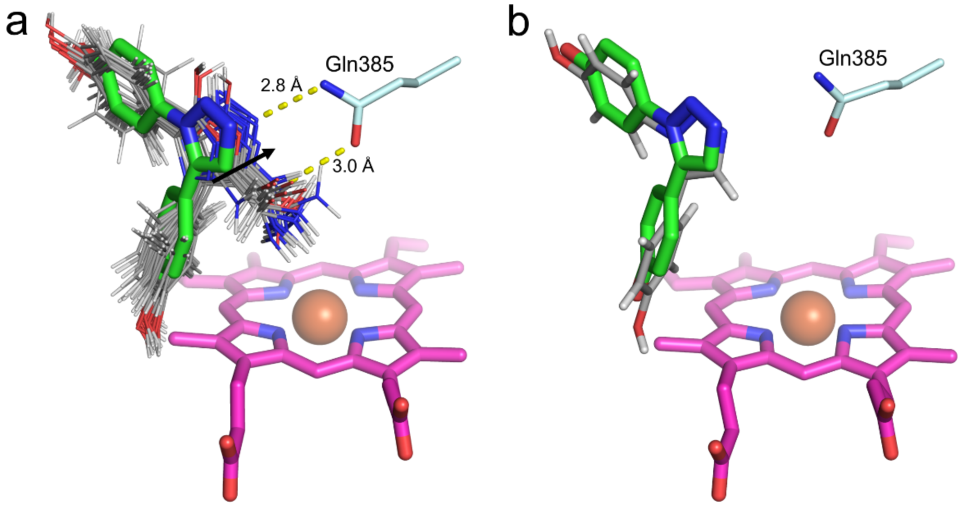

Figure S2. (**a**) Overlay of the highest ranked GOLD docking pose (grey sticks) for the biphenol analogs of **4** (see Supplementary Figure S1) screened *in silico* to promote an interaction with CYP121 Gln385. Their relative position to **4** (green sticks, PDB ID 4G2G) and the Gln385 side chain is shown. Arrow indicates increased docking proximity to Gln385. The dual H-bond interactions with Gln385 for the analogs (see structures boxed in Supplementary Figure S1) predicted to bind in closest proximity to the side chain are indicated by dashed yellow lines. (**b**) Highest scored GOLD docking pose of **4** (grey, as a control) in its position relative to **4** from the experimentally determined X-ray crystal structure (green, PDB ID 4G2G) and the Gln385 side chain.

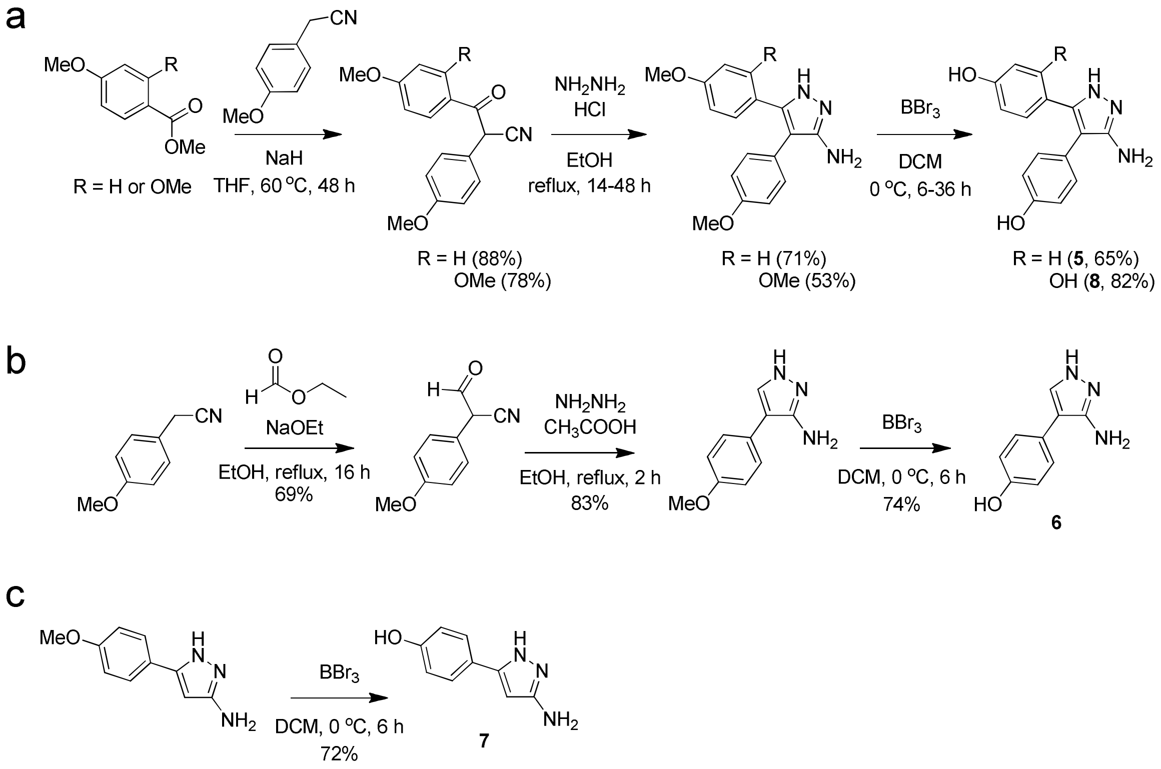

Figure S3. Synthetic route to virtual screening hit **5** (scheme **a**) and its analogs **6** (**b**) and **7** (**c**). The same route (**a**) was also used to achieve the ortho-OH derivative **8**.

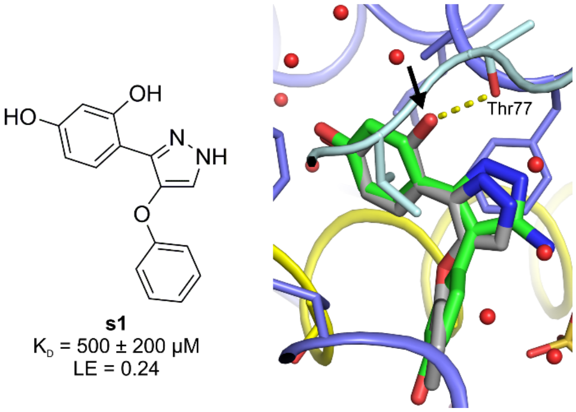

Figure S4. The comparative CYP121 binding positions of **5** (green) and the phenoxypyrazole ligand[1] **s1** (grey, PDB ID 4G48), showing their exact phenol overlap and thus the potential for adding an ortho-OH into **5** (arrow).

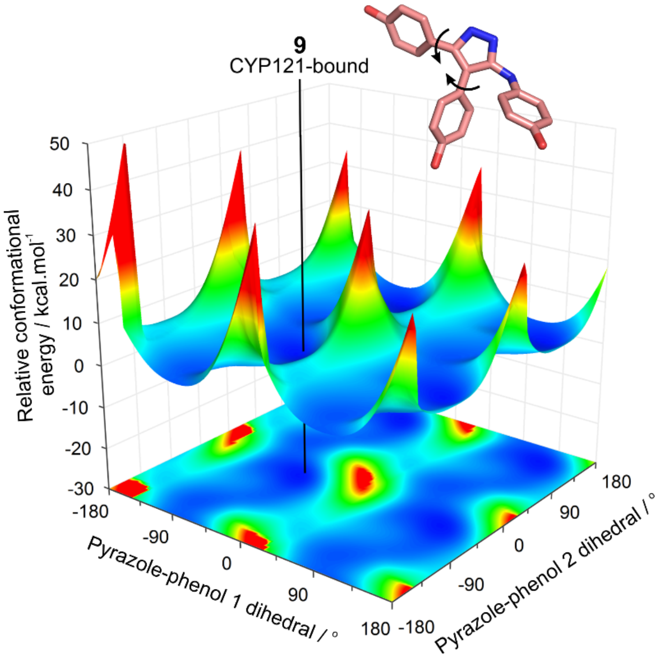

Figure S5. Computed conformational energy landscape of **9** for all rotations of its pyrazole-phenol dihedral angles (force field OPLS-2005 based methods, solvent: water). The third aminophenol was fixed relative to the pyrazole as per the CYP121-bound crystal structure in Figure 3b. The low energy dihedral positions for CYP121-bound **9** are clearly indicated.

Table S1. Macromolecule X-ray crystallography data collection and final refinement statistics

	**5**-CYP121	**6**-CYP121	**8**-CYP121	**9**-CYP121
PDB ID	4KTF	4KTJ	4KTK	4KTL
**Data collection***				
Space group	$P6_522$	$P6_522$	$P6_522$	$P6_522$
Cell dimensions				
a, b, c (Å)	78.20, 78.20, 263.19	78.03, 78.03, 265.16	77.77, 77.77, 263.60	77.79, 77.79, 264.24
α, β, γ (°)	90.0, 90.0, 120.0	90.0, 90.0, 120.0	90.0, 90.0, 120.0	90.0, 90.0, 120.0
Resolution (Å)	36.82 - 1.35 (1.42 - 1.35)	35.69 - 1.35 (1.42 - 1.35)	38.88 - 1.40 (1.48 - 1.40)	53.51 - 1.95 (2.06 - 1.95)
No. reflections (total)	283154 (42953)	238429 (32780)	165640 (22967)	139769 (10707)
No. reflections (unique)	96128 (14436)	83209 (12799)	76131 (9977)	34683 (4447)
R_{merge}	10.4 (41.4)	6.1 (36.0)	6.4 (40.2)	6.1 (8.0)
$I / \sigma I$	5.9 (2.3)	7.4 (2.1)	7.1 (2.1)	15.1 (7.5)
Completeness (%)	92.6 (96.3)	80.3 (85.6)	82.8 (75.1)	97.5 (88.4)
Multiplicity	2.9 (3.0)	2.9 (2.6)	2.2 (2.3)	4.0 (2.4)
**Refinement**				
Resolution (Å)	1.35	1.35	1.40	1.95
No. reflections	91307	79012	72290	32890
R_{work} / R_{free}	16.6 / 19.5	16.5 / 19.5	15.5 / 18.3	14.3 / 18.9
R.m.s. deviations				
Bond lengths (Å)	0.030	0.030	0.030	0.022
Bond angles (°)	2.648	2.651	2.603	2.110

*Data for the highest resolution shell are shown in parentheses

Experimental Methods

Materials

All reagents and chemicals were of analytical grade and were supplied by Sigma-Aldrich, Alfa Aesar or Maybridge. Compounds **5-8** were synthesized according to the scheme in Supplementary Figure S3.[2] Compounds **9-14** were from the DuPont HTS collection (DuPont Crop Protection USA) and were kindly provided by Dr Robert Pasteris. THF was distilled under an atmosphere of dry nitrogen from lithium aluminium hydride and calcium hydride in the presence of triphenylmethane; DCM was distilled from calcium hydride. Thin layer chromatography (TLC) was performed on glass plates coated with Merck 60 F_{254} silica and visualization was achieved by UV light or by staining potassium permanganate. Flash column chromatography was carried out using Biotage Isolera One and Biotage Isolera Four systems with UV detection at 254 and 280 nm. NMR spectra were recorded on a Bruker Avance 400 (^{1}H: 400 MHz and ^{13}C: 100 MHz), or Bruker Avance Cryo 500 (^{1}H: 500 MHz and ^{13}C: 125 MHz). Chemical shifts are quoted in ppm and are referenced to the residual non-deuterated solvent peak, and are reported (based on appearance rather than interpretation) as follows: chemical shift δ/ppm (number of protons, multiplicity, coupling constant J/Hz, assignment) [br, broad; s, singlet; d, doublet; t, triplet; q, quartet; qui, quintet; sept, septet; m, multiplet]. Infrared spectra were recorded neat on a Perkin-Elmer Spectrum One spectrometer fitted with an attenuated total reflectance (ATR) attachment with internal referencing. High-resolution mass measurements were performed on a Waters LCT Premier mass spectrometer or a Kratos Concept mass spectrometer. Low-resolution measurements were recorded on a Waters / ZQ LCMS.

Virtual screening

Docking was performed using CCDC GOLD Suite 5.1 with the X-ray crystal structure of the parent triazolylphenol fragment **1** as the protein model (PDB ID 4G47), due to its high resolution and clearly resolved/modelled active-site water network (compared with the structure of the weak merged fragment **4**). The goldscore_p450_csd template was loaded and default settings were used with the following additional parameters. All active-site waters within 5 Å of the original fragment were retained for docking. The binding site was defined with its center at the location of the fragment triazoles and a radius of 7 Å. For docking of **1** and **4** as a control, the active-site waters were configured to always on. For docking of the 31 biphenol analogs of **4**, the waters were set to toggle to allow for accommodation of the additional structural moieties within the active-site, and the Gln385 side chain was allowed flexibility using the crystal rotamer library operation with a default delta in the chi angles of $\pm 10^{o}$. Only the best solution for each ligand was kept and shown. Docking of fragment **1** was not successful in recapitulating either of the original observed fragment configurations (data not shown).

Expression and purification of Mtb CYP121

Recombinant untagged CYP121 and N-terminal His_6-tagged CYP121 were expressed and purified from the pET11a/*CYP121* and pHAT2/*CYP121* expression vectors as previously described.[1]

Isothermal titration calorimetry (ITC)

ITC binding isotherms were recorded on a MicroCal iTC_{200} microcalorimeter (GE Healthcare), integrated and fitted to estimate the K_D as previously described.[1] Titrations were initially performed with the ligand at its maximum concentration/solubility in protein buffer with 10% (v/v) DMSO, but if the resulting binding isotherm was too steep then the ligand concentration was lowered until enough injection data points were collected for a good fit (e.g. 25 mM fragment or 1 mM lead **9**).

X-ray crystallography

Crystals of untagged CYP121 were grown by sitting-drop vapor-diffusion and soaked with ligand as previously described.[1] Diffraction data sets were collected, reduced and scaled, and the structures refined as previously reported.[1] Data collection and final refinement statistics are given in Supplementary Table S1. The structures are deposited in the Protein Data Bank (http://www.rcsb.org/pdb/) under the following accession codes: **5-**CYP121: 4KTF; **6-**CYP121: 4KTJ; **8-**CYP121: 4KTK; **9-**CYP121: 4KTL. Images for presentation were rendered with PyMOL academic 1.3 (Schrödinger, Camberley, UK). A 1.35 Å dataset for analog **7** was collected, but unfortunately gave uninterpretable ligand density in the active-site (low ligand occupancy, data not shown).

In silico conformational energetic calculations

The relative conformational energy landscapes of **5** and **9** for full 360° rotations of their pyrazole-phenol dihedral angles were computed rapidly in 5° increments using a MacroModel coordinate scan (force field: OPLS-2005, solvent: water, default settings) in Schrödinger Maestro 9.0 (Schrödinger, Camberley, UK). Their pyrazole rings were constrained planar and the third aminophenol of **9** was fixed relative to the pyrazole as per the CYP121-bound crystal structure. The landscapes were plotted as a 3D map using Graphis 2.9.33 (Kylebank Software, Ayr, UK).

The global ground state conformation of the ligands was predicted rapidly by a MacroModel conformational search (method: mixed torsional/low-mode sampling, force field: OPLS-2005, solvent: water, default settings) in Schrödinger Maestro. The reported gas phase energy difference (in $kcal.mol^{-1}$) between the ligand in its predicted energy minimum conformation and that from the CYP121-bound crystal structure was calculated quantum mechanically using Jaguar optimization (QM method: DFT(b3lyp), QM basis: 6-31g**, default settings) in Schrödinger Maestro. Ligand conformations were fixed as per the crystal structure by manually constraining all the major internal dihedrals.

Organic synthesis

2,3-bis(4-methoxyphenyl)-3-oxopropanenitrile[2a]

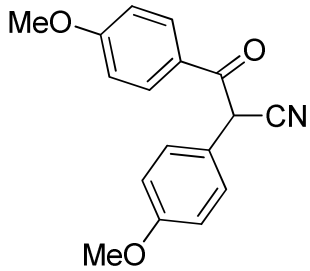

Sodium hydride (60% in mineral oil) (0.80 g, 33.6 mmol) was suspended in anhydrous THF (30 mL). 4-Methoxyphenylacetonitrile (2.28 g, 15.5 mmol) and methyl-*p*-anisate (2.84 g, 17.1 mmol) was added to the reaction and this was stirred at 60°C for 48 hours. The reaction went from a white suspension to a dark brown suspension. The reaction was cooled to room temperature and water (10 mL) was added to the reaction and the volume of the reaction was reduced to approximately 15 mL using a rotary evaporator. The reaction was extracted into DCM (3 x 50 mL). The organic phase was discarded and the aqueous layer was acidified to pH 2-3 using 10% HCl until no more precipitate was observed. The acidified aqueous layer was extracted into DCM (3 x 50 mL). The organic fractions were combined and washed with brine (3 x 10 mL) and dried over sodium sulfate. This was then filtered and the solvent removed *in vacuo* to afford the product as an orange gum which was used without further purification. Yield: 4.25 g (88%).

3-(2,4-dimethoxyphenyl)-2-(4-methoxyphenyl)-3-oxopropanenitrile

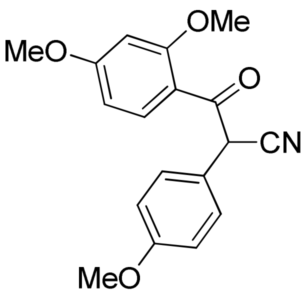

Methyl 2,4-dimethoxybenzoate (1.00 g, 5.10 mmol) and 4-methoxyphenylacetonitrile (750 mg, 5.10 mmol) were added to a suspension of 60% sodium hydride (500 mg, 12.5 mmol) in THF (25 mL). The reaction mixture was stirred at 60°C for 48 h, then water (15 mL) was added and the mixture was concentrated *in vacuo* to remove the THF. The remaining basic aqueous solution was acidified with conc. HCl and extracted with DCM (3 x 25 mL). The combined organic layers were then washed with brine (2 x 25 mL), dried over *anhydrous* Na_2SO_4 and concentrated *in vacuo*. The crude product was chromatographed using a Biotage Isolera One flash chromatography system with a Biotage SNAP cartridge (25 g silica, Biotage) and linear PET Ether/EtOAc gradient (0-100% EtOAc) as eluant, to afford the *oxopropanenitrile* (1.24 g, 3.97 mmol, 78%) as a sticky orange oil. TLC (Hexane:EtOAc, 1:1 v/v): R_f = 0.35; ^{1}H NMR (400 MHz, $CDCl_3$): δ 7.78 (d, J = 8.8 Hz, 1H), 7.26 (d, J = 8.8 Hz, 2H), 6.86 (d, J = 8.8 Hz, 2H), 6.52 (dd, J = 8.8, 2.2 Hz, 1H), 6.42 (d, J = 2.2 Hz, 1H), 5.84 (s, 1H), 3.94 (s, 3H), 3.84 (s,

3H), 3.77 (s, 3H); ^{13}C NMR (100 MHz, $CDCl_3$): δ 189.2 (C), 166.1 (C), 160.8 (C), 160.2 (C), 134.7 (CH), 130.1 (CHx2), 123.7 (C), 118.1 (C), 118.0 (C), 114.9 (CHx2), 106.6 (CH), 98.8 (CH), 56.1 (CH_3x2), 55.7 (CH_3), 49.3 (CH); MS (*m/z*): $[M + H]^+$ 312.1 (100%); HRMS (*m/z*): $[M + H]^+$ calcd. for $C_{18}H_{18}NO_4$, 312.1230; found, 312.1232.

4,5-bis(4-methoxyphenyl)-1*H*-pyrazol-3-amine[2a]

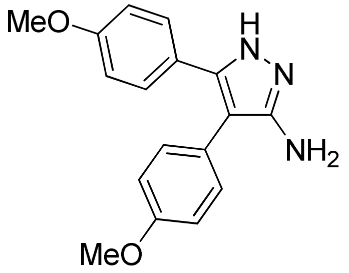

2,3-bis(4-methoxyphenyl)-3-oxopropanenitrile (2.00 g, 7.10 mmol) was dissolved in absolute ethanol (30 mL) and concentrated HCl (3.0 mL) was added slowly to the reaction. The reaction was heated to reflux and hydrazine hydrate (1.72 mL, 35.3 mmol) was added to the reaction over 5 minutes. The reaction was stirred under reflux for 14 hours. The reaction was concentrated *in vacuo* to a volume of approximately 10 mL and the reaction was diluted with water (10 mL). Saturated $NaHCO_3$ was added until the reaction was slightly basic. The reaction was extracted into EtOAc (3 x 20 mL). The organic layers were washed with brine (2 x 10 mL) and then dried over sodium sulfate. The reaction was filtered and the solvent removed *in vacuo* to afford an orange gum. This was purified on a column of flash silica gel where the product was eluted with a gradient of DCM:MeOH 10:0 to 8:2. The product was isolated as a gum which solidified on standing. Yield 1.408 g (71%) ^1H NMR (DMSO-d^6, 400 MHz): δ 11.78 (br s, 1H), 7.21 (d, *J* 8.6, 2H), 7.09 (d, *J* 8.6, 2H), 6.89 (d, *J* 8.5, 2H), 6.86 (d, *J* 8.5, 2H), 4.39 (br s, 2H), 3.75 (s, 3H), 3.73 (s, 3H); ^{13}C NMR (DMSO-d^6, 100 MHz): δ 159.0, 157.8, 131.7, 130.9, 128.7, 126.3, 124.5, 114.5, 114.2, 103.4, 55.5, 55.4; ν_{max} cm^{-1} 3217, 1622, 1532, 1513, 1489, 1202, 1179, 1020, 834; LRMS: calcd for $C_{17}H_{17}N_3O_2$ $(M+H)^+$ 296.1, found 296.1.

5-(2,4-dimethoxyphenyl)-4-(4-methoxyphenyl)-1H-pyrazol-3-amine

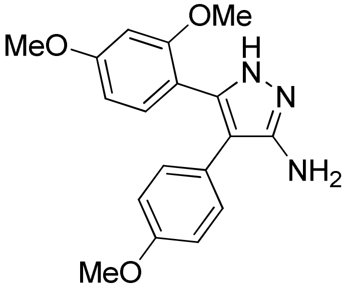

A mixture of 3-(2,4-dimethoxyphenyl)-2-(4-methoxyphenyl)-3-oxopropanenitrile (990 mg, 3.18 mmol) in EtOH (20 mL) and conc. HCl (3 mL) was brought to reflux and hydrazine hydrate (795 mg, 15.9 mmol) was added slowly over several minutes. The reaction mixture was then stirred at reflux for an additional 48 h under nitrogen, cooled to room temperature and concentrated *in vacuo* to approximately 5 mL. Water (5 mL) was added and the mixture was made weakly basic by adding saturated $NaHCO_3$ solution and then extracted with EtOAc (3 x

25 mL). The combined organic layers were washed with brine (2 x 25 mL), dried over *anhydrous* Na_2SO_4 and concentrated *in vacuo.* The crude product was chromatographed using a Biotage Isolera One flash chromatography system with a Biotage SNAP cartridge (25 g silica, Biotage) and linear DCM/MeOH gradient (0-20% MeOH) as eluant, to afford the *pyrazole* (542 mg, 1.67 mmol, 53%) as a colorless crystalline solid. TLC (DCM:MeOH, 9:1 v/v): R_f = 0.55; ^{1}H NMR (400 MHz, $CDCl_3$): δ 7.21 (d, J = 8.8 Hz, 2H), 7.07 (d, J = 8.6 Hz, 1H), 6.90 (d, J = 8.8 Hz, 2H), 6.50 (d, J = 2.4 Hz, 1H), 6.31 (dd, J = 8.6, 2.4 Hz, 1H), 3.81 (s, 6H), 3.76 (s, 3H); ^{13}C NMR (100 MHz, $CDCl_3$): δ 161.0 (C), 158.7 (C), 158.2 (C), 153.2 (C), 137.3 (C), 131.3 (CHx2), 131.1 (CH), 126.3 (C), 114.7 (CHx2), 111.8 (C), 105.8 (C), 105.2 (CH), 99.4 (CH), 56.0 (CH_3), 55.8 (CH_3), 55.6 (CH_3); MS (*m/z*): $[M + H]^+$ 326.1 (100%); HRMS (*m/z*): $[M + H]^+$ calcd. for $C_{18}H_{20}N_3O_3$, 326.1499; found, 326.1523.

4,4'-(3-amino-1*H*-pyrazole-4,5-diyl)diphenol (**5**)

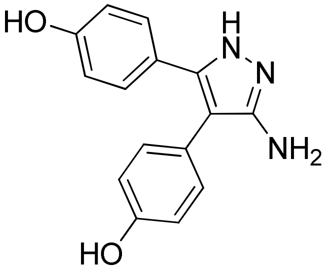

4,5-bis(4-methoxyphenyl)-1*H*-pyrazol-3-amine (0.200 g, 0.715 mmol) was suspended in anhydrous DCM (20 mL) and the reaction was cooled to 0°C. BBr_3 (1.0 M in DCM) (4.97 mL, 5.01 mmol) was added dropwise and the reaction was stirred at 0°C for 6 hours. The reaction was quenched at 0°C by addition of saturated $NaHCO_3$ (6 mL) whereupon a white precipitate formed. The reaction was extracted into ethyl acetate (3 x 20 mL) and dried over sodium sulfate. The reaction was filtered and the solvent removed *in vacuo* to afford the crude product as an off-white gum. The crude product was placed onto a column of flash silica gel where it was eluted with DCM:MeOH 1:0 to 4:1 whereupon the product was isolated as a white solid. Yield 0.124 g (65%) ^{1}H NMR (DMSO-d^6, 400 MHz): δ 11.64 (br s, 1H), 9.51 (br s, 1H), 9.30 (br s, 1H), 7.11 (d, J 8.5, 2H), 6.97 (d, J 8.5, 2H), 6.72 (d, J 8.6, 2H), 6.67 (d, J 8.6, 2H), 4.38 (br s, 2H); ^{13}C NMR (DMSO-d^6, 100 MHz): δ 157.26, 155.86, 130.9, 128.7, 124.6, 115.88, 115.6, 103.9 (some of the quaternary peaks have been masked by other peaks in the aromatic region); ν_{max} cm^{-1} 3121 (br OH), 1613, 1591, 1511, 1491, 1426, 1349, 1262, 1220, 1170, 819; LRMS: calcd for $C_{15}H_{13}N_3O_2$ $(M+H)^+$ 268.1, found 268.1 HRMS (ESI): calcd for $C_{15}H_{13}N_3O_2$ $(M+H)^+$ 268.1087, found 268.1106.

4-(3-amino-4-(4-hydroxyphenyl)-1H-pyrazol-5-yl)benzene-1,3-diol (**8**)

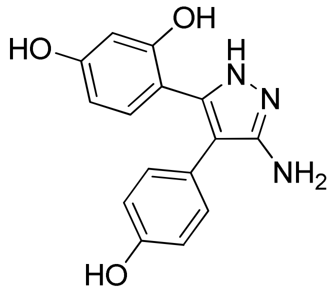

A mixture of 5-(2,4-dimethoxyphenyl)-4-(4-methoxyphenyl)-1H-pyrazol-3-amine (250 mg, 0.769 mmol) in DCM (10 mL) was cooled to 0°C and 7.7 mL of 1M boron tribromide in DCM was added dropwise over 5 min. The reaction mixture was stirred at 0°C for an additional 36 h under nitrogen and then quenched by pouring into 30 mL of saturated $NaHCO_3$ solution. The DCM was removed *in vacuo* and the aqueous layer extracted with EtOAc (2 x 25 mL). The combined organic layers were washed with H_2O (2 x 25 mL) and brine (1 x 25 mL), dried over *anhydrous* Na_2SO_4 and concentrated *in vacuo* to afford the *phenol* (178 mg, 0.628 mmol, 82%) as a cream powder. TLC (DCM:MeOH, 8:2 v/v): R_f = 0.35; ^{1}H NMR (400 MHz, MeOD): δ 7.08 (d, *J* = 8.5 Hz, 2H), 6.86 (d, *J* = 8.5 Hz, 1H), 6.78 (d, *J* = 8.5 Hz, 2H), 6.33 (d, *J* = 2.2 Hz, 1H), 6.10 (dd, *J* = 8.5, 2.2 Hz, 1H); ^{13}C NMR (100 MHz, MeOD): δ 159.4 (C), 157.9 (C), 157.2 (C), 151.0 (C), 143.0 (C), 132.1 (CHx2), 131.3 (CH), 125.8 (C), 116.6 (CHx2), 110.5 (C), 107.5 (CH), 105.5 (C), 103.7 (CH); MS (*m/z*): $[M + H]^+$ 284.1 (100%); HRMS (*m/z*): $[M + H]^+$ calcd. for $C_{15}H_{14}N_3O_3$, 284.1030; found, 284.1036.

2-(4-methoxyphenyl)-3-oxopropanenitrile[2b]

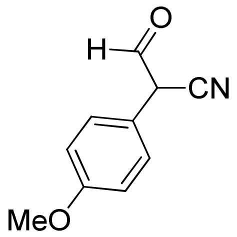

A solution of sodium ethoxide was prepared by dissolving sodium (0.273 g, 11.9 mmol) in absolute ethanol (15 mL). The mixture was cooled to 0°C and 4-methoxyphenylacetonitrile (1.58 g, 10.8 mmol) was added and the reaction was stirred for five minutes. Ethyl formate (2.00 g, 27.0 mmol) was added to the reaction mixture and this was stirred under reflux for 16 hours. The reaction was cooled to room temperature and the solvent removed *in vacuo* to afford an off-white solid. The residue was dissolved in water (10 mL) and the pH adjusted to 3-4 using 5% HCl. The mixture was extracted with diethyl ether (3 x 20 mL). The combined organic layers were combined and washed with brine (1 x 15 mL) and the organic layer was dried over sodium sulfate. The mixture was filtered and the solvent removed *in vacuo* to afford the product as an orange oil which was used without further purification. Yield 1.301 g (69%).

4-(4-methoxyphenyl)-1*H*-pyrazol-3-amine[2b]

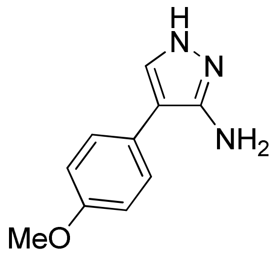

2-(4-methoxyphenyl)-3-oxopropanenitrile (1.30 g, 7.42 mmol) was dissolved in absolute ethanol (10 mL). Glacial acetic acid (1 mL) was added to the reaction followed by hydrazine hydrate (0.719 mL, 14.8 mmol) and this was heated under reflux for two hours. The reaction was cooled to room temperature and was concentrated *in vacuo.* Water (10 mL) was added to the reaction mixture followed by diethyl ether (20 mL). The organic layer was separated and washed with water (10 mL). The organic layer was dried over sodium sulfate, filtered and then concentrated to afford the crude product which was used without further purification. Yield 1.16 g (83%) ^{1}H NMR ($CDCl_3$, 400 MHz): δ 7.48 (s, 1H), 7.33 (d, J 8.8, 2H), 6.95 (d, J 8.8, 2H), 4.06 (br s, 2H), 3.82 (s, 3H); ^{13}C NMR ($CDCl_3$, 100 MHz): δ 158.6, 128.9, 128.4, 128.0, 114.6, 108.2, 55.4; As this material was not purified the 1H-NMR spectrum contained approximately 10% of impurities.

4-(3-amino-1*H*-pyrazol-4-yl)phenol (**6**)

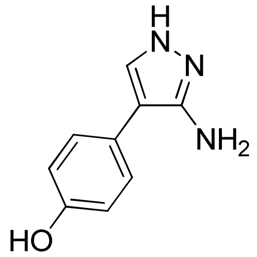

4-(4-methoxyphenyl)-1*H*-pyrazol-3-amine (0.085 g, 0.449 mmol) was suspended in anhydrous DCM (10 mL) and the reaction was cooled to 0°C. BBr_3 (1.0 M in DCM) (2.21 mL, 2.24 mmol) was added dropwise to the reaction and the reaction was stirred at 0°C for 8 hours. The reaction was quenched at 0°C by addition of saturated $NaHCO_3$ (6 mL) whereupon a white precipitate formed. The reaction was extracted into ethyl acetate (3 x 15 mL). This was dried over sodium sulfate. The reaction was filtered and the solvent removed *in vacuo* to afford the crude product as an off-white gum. The crude product was placed onto a column of flash silica gel where it was eluted with DCM:MeOH 1:0 to 2:1 whereupon the product was isolated as a white solid. Yield 0.054 g (74%); ^{1}H NMR (DMSO-d^6, 500 MHz): δ 9.17 (s, 1H), 7.49 (s, 1H), 7.25 (d, J 8.5, 2H), 6.71 (d, J 8.5, 2H); ^{13}C NMR (DMSO-d^6, 125 MHz): δ 155.2, 149.3, 129.5, 127.3, 125.1, 115.8, 106.5; ν_{max} cm^{-1} 3122 (br OH), 1596, 1562, 1510, 1490, 1427, 1364, 1218, 1177, 1005, 815; LRMS: calcd for $C_9H_9N_3O$ $(M+H)^+$ 176.0, found 176.1 HRMS (ESI): calcd for $C_9H_9N_3O$ $(M+H)^+$ 176.0825, found 176.0842.

4-(3-amino-1*H*-pyrazol-5-yl)phenol (**7**)

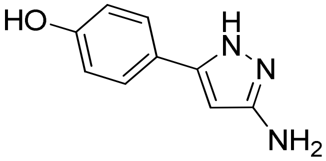

5-(4-methoxyphenyl)-1*H*-pyrazol-3-amine (0.16 g, 0.84 mmol) was suspended in anhydrous DCM (16 mL) and the reaction was cooled to 0°C. BBr_3 (1.0 M in DCM) (4.19 mL, 4.72 mmol) was added dropwise to the reaction and the reaction was stirred at 0°C for 6 hours. The reaction was quenched at 0°C by addition so saturated NaHCO3 (6 mL) whereupon a white precipitate formed. The reaction was extracted into ethyl acetate (3 x 20 mL) and dried over sodium sulfate. The reaction was filtered and the solvent removed *in vacuo* to afford the crude product as an off-white gum. The crude product was placed onto a column of flash silica gel where it was eluted with DCM:MeOH 1:0 to 2:1 whereupon the product was isolated as a white solid. Yield 0.105 g (72%) ^{1}H NMR (DMSO-d^{6}, 400 MHz): δ 9.29 (v br s, 1H), 7.42 (d, J 8.6, 2H), 6.74 (d, J 8.6, 2H), 5.60 (s, 1H), 4.62 (br s, 2H); ^{13}C NMR (DMSO-d^{6}, 100 MHz): δ 157.2, 153.9, 145.2, 126.5, 123.3, 115.8, 87.3; ν_{max} cm^{-1} 3123 (br OH), 1613, 1593, 1512, 1485, 1426, 1231, 1176, 1010, 822; LRMS: calcd for $C_9H_9N_3O$ (M+H)$^{+}$ 176.0, found 176.1 HRMS (ESI): calcd for $C_9H_9N_3O$ (M+H)$^{+}$ 176.0825, found 176.0856.

References

[1] S. A. Hudson, K. J. McLean, S. Surade, Y. Q. Yang, D. Leys, A. Ciulli, A. W. Munro, C. Abell, *Angew. Chem. Int. Ed. Engl.* **2012**, *51*, 9311.

[2] a) D. R. Compton, S. B. Sheng, K. E. Carlson, N. A. Rebacz, I. Y. Lee, B. S. Katzenellenbogen, J. A. Katzenellenbogen, *J. Med. Chem.* **2004**, *47*, 5872; b) B. T. Gregg, D. O. Tymoshenko, D. A. Razzano, M. R. Johnson, *J. Comb. Chem.* **2007**, *9*, 507.
